# Plasma Humanin and Non-Coding RNAs as Biomarkers of Endothelial Dysfunction in Rheumatoid Arthritis: A Pilot Study

**DOI:** 10.3390/ncrna11010005

**Published:** 2025-01-14

**Authors:** Donatella Coradduzza, Sara Cruciani, Biagio Di Lorenzo, Maria Rosaria De Miglio, Angelo Zinellu, Margherita Maioli, Serenella Medici, Gian Luca Erre, Ciriaco Carru

**Affiliations:** 1 Department of Biomedical Sciences, University of Sassari, 07100 Sassari, Italy; sara.cruciani@outlook.com (S.C.); bdilorenzo@uniss.it (B.D.L.); azinellu@uniss.it (A.Z.); mmaioli@uniss.it (M.M.); carru@uniss.it (C.C.); 2 Department of Medicine, Surgery and Pharmacy, University of Sassari, 07100 Sassari, Italy; demiglio@uniss.it (M.R.D.M.); glerre@uniss.it (G.L.E.); 3 Department of Chemical, Physical, Mathematical and Natural Sciences, University of Sassari, 07100 Sassari, Italy; sere@uniss.it

**Keywords:** biomarkers, humanin, miRNA, long non-coding RNA, cardiovascular risk, endothelial dysfunction, rheumatoid arthritis

## Abstract

**Background:** Rheumatoid arthritis (RA) is a chronic autoimmune disorder associated with an increased risk of cardiovascular disease (CVD), largely driven by peripheral endothelial dysfunction (ED). Humanin, a mitochondrial-derived peptide, has been suggested to play a protective role in endothelial function. However, the relationship between Humanin levels and ED in RA, as well as the interaction between Humanin and non-coding RNAs such as Long Non-Coding RNA GAS5, microRNA-21 (miR-21), and microRNA-103 (miR-103), remains unclear. **Objective:** This study aimed to investigate the relationship between circulating Humanin levels, non-coding RNAs (GAS5, miR-21, miR-103), and endothelial dysfunction (ED) in patients with RA. Additionally, we explored the correlation between Humanin expression and specific non-coding RNAs (GAS5, miR-21, and miR-103) to better understand their potential role in vascular health. **Methods:** Peripheral ED was assessed using flow-mediated pulse amplitude tonometry, with Ln-RHI values <0.51 indicating dysfunction. Humanin levels, GAS5, miR-21, and miR-103 were measured in RA patients. Univariate and multivariate analyses were conducted to determine the relationship between these biomarkers and ED. Kaplan–Meier survival analysis and ROC curve analysis were used to assess the prognostic value of Humanin. **Results:** Higher Humanin levels were significantly associated with better endothelial function (OR = 0.9774, *p* = 0.0196). Kaplan–Meier analysis demonstrated that higher Humanin levels correlated with improved survival (*p* < 0.0001). The non-coding RNAs (GAS5, miR-21, and miR-103) did not show significant associations with ED. **Conclusions:** Humanin is a potential protective biomarker for endothelial dysfunction and survival in RA patients. Further research is needed to explore the interaction between Humanin and non-coding RNAs in the context of vascular health.

## 1. Introduction

Rheumatoid arthritis (RA) is a chronic autoimmune disease characterized by persistent and systemic inflammation, which leads to joint destruction and significantly increases the risk of mortality [1,2]. In addition to musculoskeletal manifestations, RA is associated with systemic complications, including atherosclerotic cardiovascular disease (CVD), closely linked to chronic systemic inflammation and dysregulated immune responses [3,4]. Patients with RA often develop peripheral endothelial dysfunction (ED) early in the disease course, which over time predisposes them to fatal cardiovascular events and sudden death [5,6]. The loss of the endothelium’s ability to regulate vascular tone, maintain blood flow, and prevent thrombosis makes this an important area of investigation in RA [7,8,9]. However, the mechanisms underlying ED and the increased cardiovascular disease risk in RA are not fully explained by traditional cardiovascular risk factors, suggesting the involvement of additional metabolic pathways [10,11]. Among the various metabolites studied, Humanin (HN), a highly conserved mitochondrial-derived peptide, has been shown to protect endothelial cells from oxidative stress and apoptosis induced by oxidized low-density lipoprotein (LDL) [12]. Circulating levels of HN are associated with preserved coronary endothelial function, emphasizing its critical role in vascular health regulation [13]. HN exerts its protective effects by interacting with members of the Bcl-2 family, preventing apoptosome formation and inhibiting the translocation of pro-apoptotic proteins to the mitochondria, thereby protecting various cell types, including neurons, pancreatic β-cells, testicular germ cells, and endothelial cells, from oxidative stress and apoptosis [14]. Additionally, HN supports mitochondrial biogenesis, further promoting cellular survival. Given the critical role that oxidative stress plays in both the pathogenesis of RA and the development of endothelial dysfunction, investigating the relationship between circulating Humanin levels, non-coding RNAs (GAS5, miR-21, miR-103), and endothelial dysfunction (ED) in patients with RA is of great interest [15]. Although the exact relationship remains unclear, it is plausible that HN may exert a protective role in attenuating endothelial dysfunction in this patient population. Furthermore, the exploration of potential correlations with non-coding RNAs, such as Long Non-Coding RNA Growth Arrest-Specific 5 (GAS5), microRNA-21 (miR-21), and microRNA-103 (miR-103), may provide additional insights into the molecular mechanisms underlying vascular function regulation in RA [16,17,18]. The potential interactions between HN and these non-coding RNAs may involve the regulation of common target genes, competition for mRNA binding sites, or the activation of synergistic pathways aimed at protecting cells and modulating stress responses [19,20,21,22]. GAS5, known for its role in controlling apoptosis and immune responses, along with miR-21 and miR-103, involved in apoptosis regulation and metabolism, may significantly influence gene expression and cellular homeostasis relevant to endothelial function [23,24,25]. The primary aim of this study is to investigate the relationship between Humanin levels and peripheral endothelial dysfunction in patients with rheumatoid arthritis, with a secondary focus on correlating GAS5, miR-21, and miR-103 expression levels. Peripheral ED will be assessed using flow-mediated pulse amplitude tonometry, with logarithmic reactive hyperemia index (Ln-RHI) values serving as a quantitative measure of endothelial function. Ln-RHI values below 0.51 will indicate the presence of ED, allowing for the correlation with Humanin levels and other molecular markers [26]. The findings of this study could improve the understanding of the pathophysiological mechanisms underlying the increased cardiovascular risk in RA and contribute to the development of new therapeutic strategies targeting endothelial dysfunction in this patient population.

## 2. Results

This study included a total of 90 RA patients diagnosed, of whom 71 were male (mean age: 66.13 ± 10.53 years) and 19 were female (mean age: 70.05 ± 8.90 years). The overall mean age of the participants was 66.96 ± 10.28 years. The primary objective of this study was to evaluate the relationship between circulating Humanin levels, Long Non-Coding RNA GAS5, microRNA-21 (miR-21), microRNA-103 (miR-103), and peripheral endothelial dysfunction (ED) in these patients.

Patients were stratified into groups based on cardiovascular disease risk, using the EULAR SCORE-2 algorithm. The stratification resulted in the following groups: low cardiovascular risk (RA-LR), medium cardiovascular risk (RA-MR), high cardiovascular risk (RA-HR), and those with adverse cardiovascular outcomes (RA-D). Baseline characteristics are summarized in Table 1.

### 2.1. miRNA Expression

**Figure 1:**
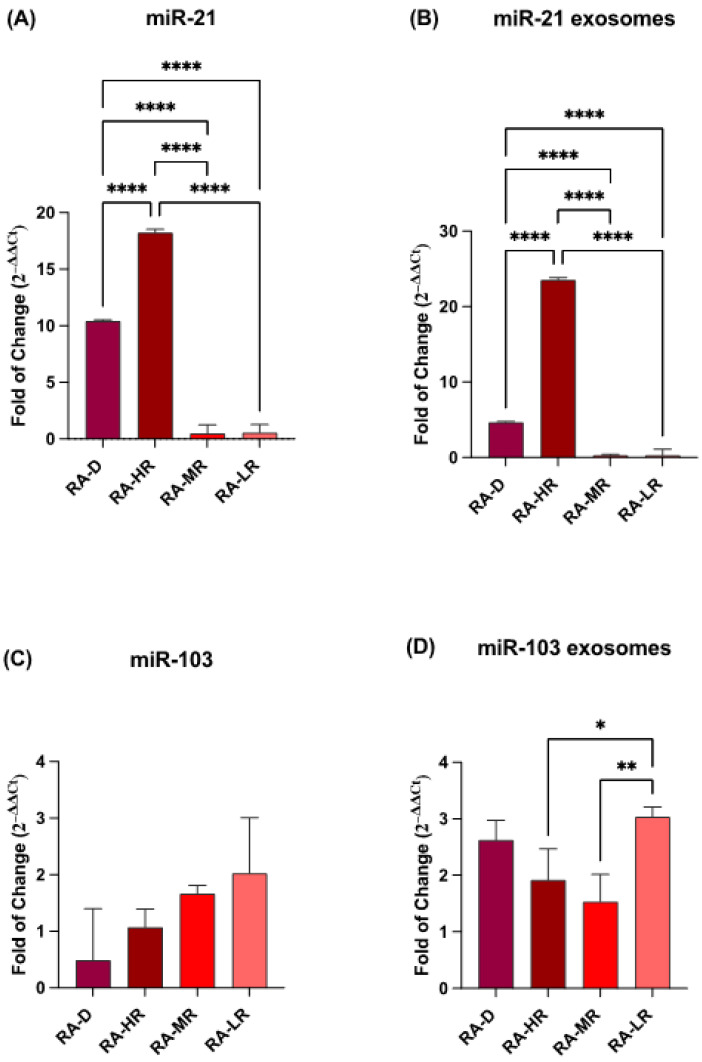
miRNA expression in plasma and exosomes. The expression of miR-21 Panels (**A**) and (**B**) and miR-103 Panels (**C**) and (**D**) was evaluated in plasma and exosomes. mRNA levels were normalized to U6snRNA. The data are presented as the mean ± SD relative to the control (* *p* ≤ 0.05, ** *p* ≤ 0.01, and **** *p* ≤ 0.0001).

The expression of miR-21 and miR-103 was assessed in both plasma and exosomes of the RA patients. As illustrated in Figure 1, miR-21 (Panels A and B) was significantly upregulated in RA-D patients and those with a high cardiovascular risk (RA-HR). Conversely, miR-103 showed a different expression pattern, with significantly elevated levels in the plasma of low-cardiovascular-risk (RA-LR) patients, as shown in Figure 1.

### 2.2. Lnc-RNA GAS5 Expression

**Figure 2:**
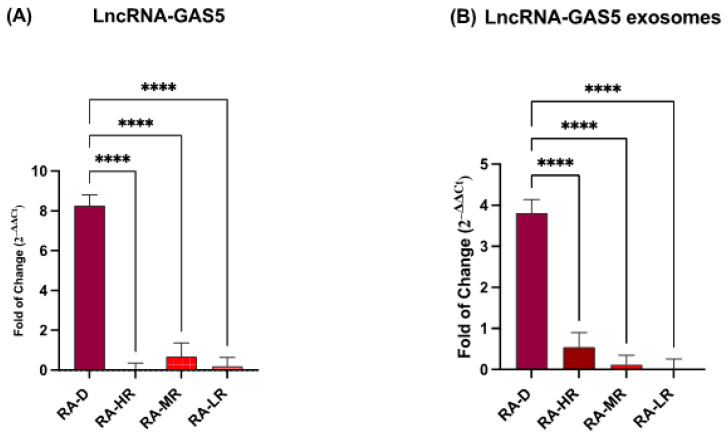
Lnc-RNA GAS5 expression in plasma (**A**) and (**B**). Expression levels were normalized to Glyceraldehyde-3-Phosphate-Dehydrogenase (GAPDH). The data are presented as the mean ± SD relative to the control (**** *p* ≤ 0.0001).

Figure 2 shows the expression levels of Long Non-Coding RNA GAS5 in the plasma of RA patients. GAS5 expression was significantly increased in both the plasma and exosomes of RA-D patients, as shown in Figure 2.

### 2.3. Humanin Levels in Plasma Samples

**Figure 3:**
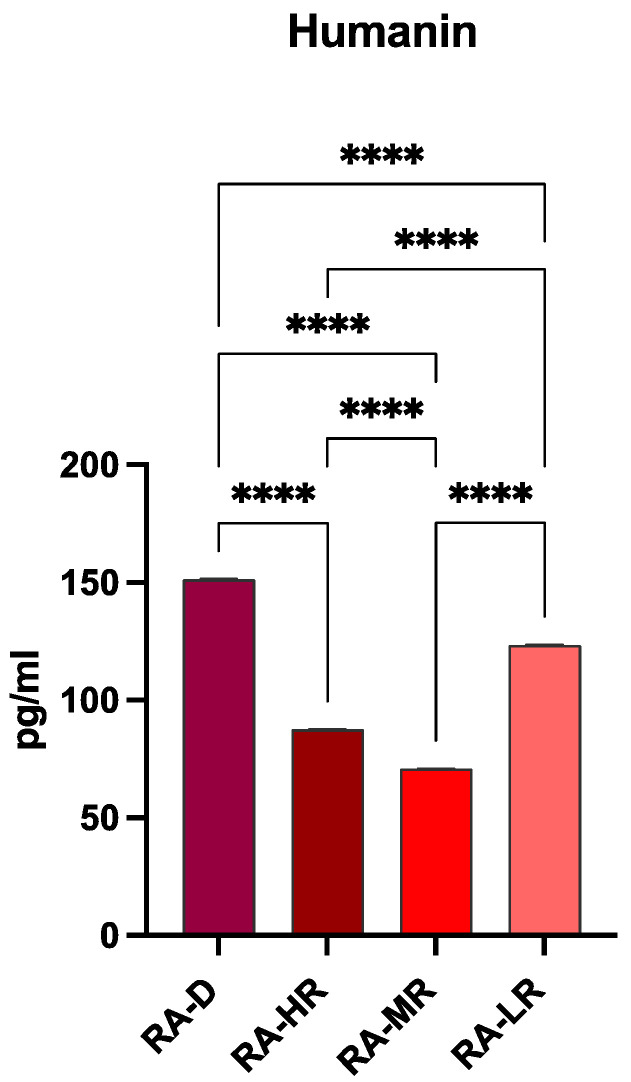
Humanin levels in plasma were determined by ELISA. Data are presented as mean ± SD relative to control (**** *p* ≤ 0.0001).

Humanin secretion was quantified by using an ELISA in plasma samples. As shown in Figure 3, RA-VD patients had significantly higher levels of circulating Humanin compared to the other patient groups.

### 2.4. Peripheral Endothelial Dysfunction

Peripheral ED was assessed using flow-mediated pulse amplitude tonometry, with the logarithmic reactive hyperemia index (Ln-RHI) serving as a measure of endothelial function. Ln-RHI values below 0.51 were considered indicative of ED.

### 2.5. Multivariate Logistic Analysis

The results involve a multivariate logistic regression analysis to evaluate the association between age, sex, SCORE2_EULAR, circulating Humanin levels, and non-coding RNAs (miR-21, miR-103, and GAS5, both free and exosomal), with endothelial function as the primary outcome. This analysis was conducted to control for confounding factors and assess the independent prognostic power of each biomarker. The analysis demonstrated a significant association between circulating Humanin levels and ED at 0.9774 (9-fold higher Humanin levels were protective against peripheral endothelial dysfunction (ED)). For the non-coding RNAs, neither miR-21 nor **miR-103 showed a significant association. GAS5 in exosomes showed a trend toward an association with ED (OR = 1.0976, 95% CI: 0.9111 to 1.3223, *p* = 0.327), although this result did not reach significance. To ensure robustness, the independent prognostic power of each biomarker was separately assessed with adjustments for confounders such as age, sex, and SCORE2_EULAR. The findings underscore Humanin’s unique and statistically significant association with endothelial function, positioning it as a potentially valuable biomarker in assessing cardiovascular risk in RA patients, as shown in Table 2.

### 2.6. ROC Curve Analysis

**Figure 4:**
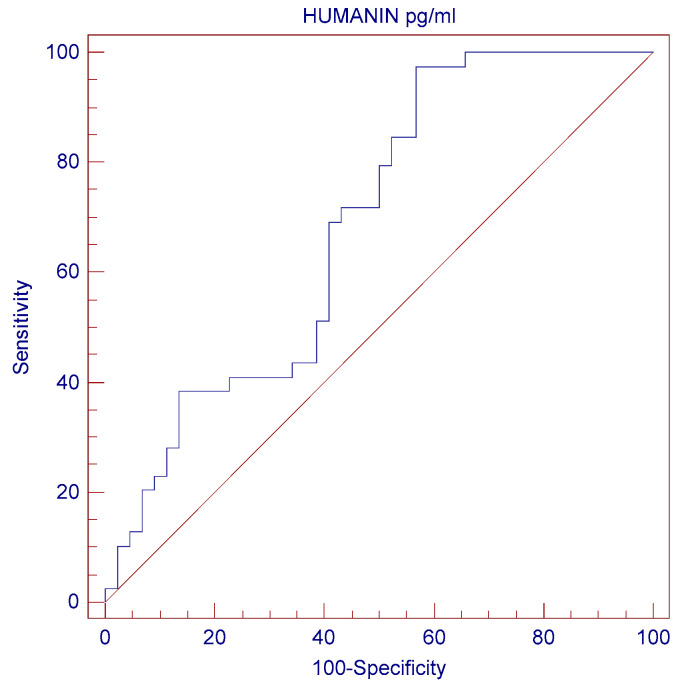
ROC curve analysis for Humanin’s predictive value for ED.

To further explore Humanin’s predictive value for ED, a ROC curve analysis was performed (Table 3 and Figure 4). The area under the ROC curve (AUC) was 0.685897 (95% CI: 0.574686 to 0.783392, *p* = 0.0015), indicating a moderate predictive ability of Humanin levels for ED. The optimal cutoff for Humanin levels was 124.44 pg/mL, with a Youden index of 0.4062. This threshold was associated with a higher likelihood of ED in patients with Humanin levels below the cutoff, as shown in Table 3 and Figure 4.

### 2.7. Kaplan-Meier Survival Analysis

**Figure 5:**
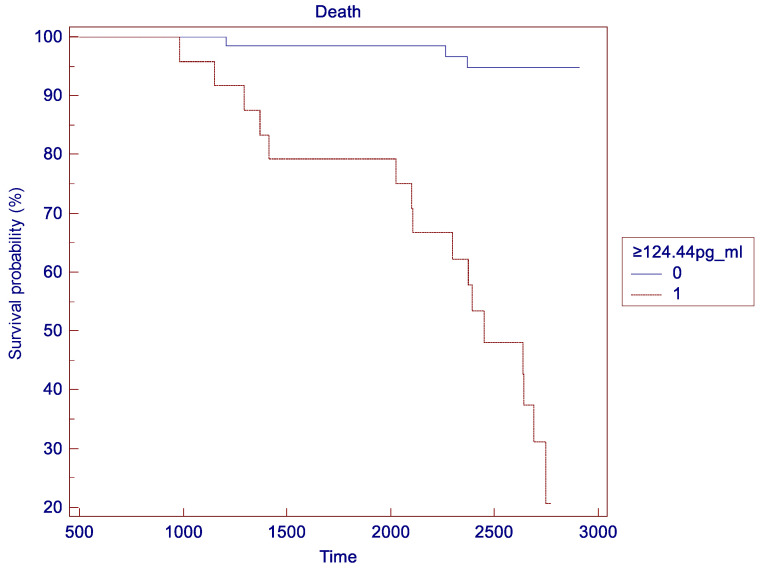
Kaplan–Meier survival analysis to evaluate the prognostic value of Humanin levels for survival outcomes. 0 = patients with serum Humanin concentration < 124.44pg/mL; 1 = patients with serum Humanin concentration ≥ 124.44pg/mL.

Kaplan–Meier survival analysis was conducted to evaluate the prognostic value of Humanin levels for survival outcomes (Figure 5 and Table 4 and Table 5). Patients were divided into two groups based on the identified cutoff for Humanin levels (124.44 pg/mL). The log-rank test revealed a highly significant difference in survival between the two groups (chi-square = 42.56, *p* < 0.0001). Patients with higher Humanin levels (≥124.44 pg/mL) showed significantly better endothelial function and higher survival rates than those with lower Humanin levels (<124.44 pg/mL).

### 2.8. Hazard Ratio for Mortality

**Figure 6:**
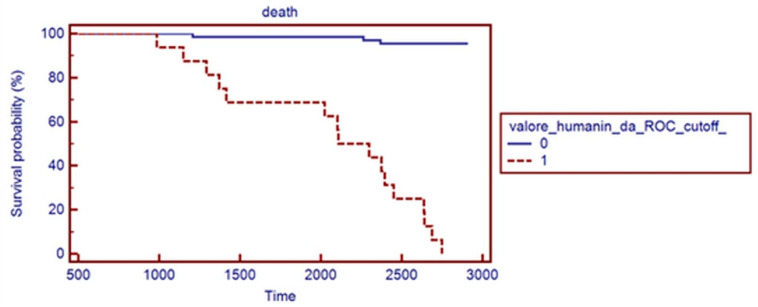
The Kaplan–Meier survival curve showing the survival probabilities for the two groups based on the ROC-determined Humanin levels. Group 0 (blue): Higher survival probability. Group 1 (red dashed): Lower survival probability. The number at risk at different time points is detailed in Table 5.

The hazard ratio (HR) for mortality based on Humanin levels was 18.66 (95% CI: 6.33 to 55.02, *p* < 0.0001), indicating a substantially higher rate of mortality for patients with Humanin levels below the cutoff. This finding underscores the potential of Humanin as a biomarker for both peripheral endothelial dysfunction and overall survival in RA patients, as shown in Figure 6.

## 3. Discussion

There is promising evidence regarding the potential role of Humanin in cardiovascular health. Humanin, an 11-amino acid peptide, appears to possess cellular protective properties, reducing oxidative stress in blood vessels. Although the exact mechanism of action of Humanin is still under investigation, it is believed that this molecule involves both extracellular receptors and intracellular interactions with a variety of proteins, exhibiting broad protective effects across different cellular models. The results of this study provide valuable insights into the relationship between Humanin levels and peripheral endothelial dysfunction (ED) in RA patients, while also exploring the role of non-coding RNAs, including GAS5, miR-21, and miR-103. Our findings highlight the critical role of Humanin as a protective factor for endothelial function and its potential as a prognostic biomarker for cardiovascular risk and survival in RA patients. Specifically, higher circulating levels of Humanin were associated with better endothelial function, and this protective effect remained significant even after adjusting for key variables such as age, sex, and the cardiovascular risk SCORE2_EULAR score. This suggests that Humanin may play an important role in maintaining vascular health in RA patients. Given that endothelial dysfunction is a key factor contributing to the development of atherosclerotic cardiovascular disease (CVD), which is highly prevalent in RA, these findings underscore the potential importance of Humanin in modulating cardiovascular outcomes in this population. These results are consistent with previous studies that have demonstrated Humanin’s cytoprotective properties, particularly its ability to mitigate oxidative stress and apoptosis in endothelial cells. In contrast, the analysis of non-coding RNAs (GAS5, miR-21, and miR-103) did not reveal significant correlations with endothelial function in this cohort. Although GAS5 showed a trend toward a protective role, it did not reach statistical significance, suggesting that further investigation with larger sample sizes would be appropriate. Additionally, the Kaplan–Meier survival analysis revealed a strong association between higher Humanin levels and improved survival outcomes in RA patients. Patients with Humanin levels above the identified threshold of 124.44 pg/mL showed significantly better endothelial function and higher survival rates. The ROC curve analysis supported these results, demonstrating the moderate predictive power of Humanin for identifying patients with endothelial dysfunction, indicating its potential as a biomarker for both endothelial health and overall prognosis in RA patients. Furthermore, the hazard ratio for mortality further highlighted the prognostic value of Humanin, suggesting that low Humanin levels could serve as an early indicator of deteriorating vascular function and increased mortality risk. These findings reinforce Humanin’s potential as a key biomarker for predicting cardiovascular outcomes and survival in RA patients.

It is clear that there are present limitations related to the variability in drug histories among the patient groups, including differences in medication types and doses. While adjustments were made for cardiovascular risk factors and disease-related variables, unstandardized treatment regimens may have influenced the biomarkers and endothelial function outcomes assessed in this study. Future research should prioritize more controlled settings to evaluate the specific impacts of medication regimens on vascular health and biomarker expression.

In conclusion, this study highlights Humanin as a key regulator of endothelial function and a potential biomarker for predicting cardiovascular outcomes and mortality in RA patients, contributing to a deeper understanding of the molecular mechanisms linking RA, endothelial dysfunction, and cardiovascular risk in this vulnerable patient population. Given the chronic inflammatory state in RA, which contributes to both disease progression and cardiovascular complications, Humanin’s role in counteracting these effects could have clinical implications. These findings contribute to a deeper understanding of the molecular mechanisms linking RA, endothelial dysfunction, and cardiovascular risk in this vulnerable patient population.

## 4. Materials and Methods

### 4.1. Subjects

This study included a total of 90 patients diagnosed with rheumatoid arthritis, comprising 71 males (mean age: 66.13 ± 10.53 years) and 19 females (mean age: 70.05 ± 8.90 years), with an overall mean age of 66.96 ± 10.28 years. Patients were classified based on the 2010 European Alliance of Associations for Rheumatology (EULAR) and American College of Rheumatology (ACR) criteria for RA diagnosis [27]. All participants were prospectively enrolled in the auxiliary study of the Endothelial Dysfunction Evaluation for Coronary Heart Disease Risk Estimation in Rheumatoid Arthritis (EDRA study; ClinicalTrials.gov: NCT02341066). The inclusion and exclusion criteria for the Bio-RA and EDRA studies have been previously published [28]. Briefly, eligible patients were adults diagnosed with RA, meeting the EULAR/ACR criteria, and were either at risk or undergoing evaluation for cardiovascular disease (CVD) [29]. Patients were excluded if they had comorbid conditions that could independently affect endothelial function or if they were unable to comply with study procedures. The study protocol was approved by the Ethics Committee of ASL 1 Sassari (Italy) (approval numbers 2126/CE-2015 and 2219/CE-2015). All study procedures were conducted in compliance with the Declaration of Helsinki. Written informed consent was obtained from each participant prior to their inclusion in the study, ensuring that all participants were fully aware of the study’s purpose, procedures, and potential risks.

### 4.2. Quantification of miR-21 and miR-103 in Plasma and Exosomes

The TaqMan^®^ MicroRNA Reverse Transcription Kit (Thermo Fisher Scientific, Grand Island, NY, USA) followed by qPCR was used to evaluate the level of expression of hsa-miR-21-5p (miR-21) and hsa-miR-103a-3p (miR-103) in plasma samples and exosomes. RNA extraction was performed using the miRNeasy Serum/Plasma Kit (Qiagen, Hilden, Germany) and the miRNeasy Mini Kit (Qiagen, Hilden, Germany) according to the manufacturer’s instructions. The Ct values for each miRNA were normalized to U6snRNA. The miRNA sequences are shown in Table 6.

### 4.3. Gene Expression Analysis

Total RNA was extracted from plasma and exosomes using the miRNeasy Serum/Plasma Kit (Qiagen, Hilden, Germany) and the miRNeasy Mini Kit (Qiagen, Hilden, Germany), respectively. The RNA of each sample was quantified by using the NanoDrop™ One/OneC Microvolume UV-Vis spectrophotometer (Thermo Fisher Scientific, Grand Island, NY, USA) and then reverse-transcribed using the High-Capacity cDNA Reverse Transcription Kit (Applied Biosystems, Waltham, MA, USA, Thermo Fisher Scientific, Grand Island, NY, USA). SYBR™ Green PCR Master Mix (Applied Biosystems, Waltham, MA, USA, Thermo Fisher Scientific, Grand Island, NY, USA) was used for the qPCR in a CFX Thermal Cycler (Bio-Rad, Hercules, CA, USA). Target Ct values of each sample for LncRNA-GAS5 were normalized to a reference gene, hGAPDH. The primers used (Thermo Fisher Scientific, Grand Island, NY, USA) are described in Table 7.

### 4.4. ELISA

Humanin levels were measured in plasma samples by using an ELISA with the Human Humanin ELISA Kit (Assay Genie, Dublin, Ireland) following the manufacturer’s instructions. A total of 100 μM of standards and samples was added to each well and incubated at 37 °C for 90 min. Then, Biotin–streptavidin incubation was performed at 37 °C for 90 min. Finally, a TMB substrate was incubated in the dark for 20 min, and then the absorbance was read at 450 nm using a microplate reader (Akribis Scientific, Common Farm, Frog Ln, Knutsford WA16 0JG, Great Britain, UK). Each sample was assayed in duplicate, and the values were expressed as the mean ± SD of 2 measures per sample. The concentration of the samples was determined from the standard curve.

### 4.5. Flow-Mediated Pulse Amplitude Tonometry (PAT)

Fasting RA patients were examined using flow-mediated pulse amplitude tonometry (PAT) to assess peripheral endothelial dysfunction (ED). The assessments were conducted in a quiet, temperature-controlled room to minimize environmental influences. Pulsatile volume changes in the digital artery were measured using probes placed on the middle finger of both hands which consisted of expandable compartments. These volume changes were registered as pulse amplitude signals and recorded by the EndoPAT 2000 system (Itamar Medical Inc., Caesarea, Israel). The PAT procedure began with a 5 min baseline measurement period, during which the digital artery’s baseline pulse amplitude was recorded. Following this, the blood flow in the digital artery was occluded by inflating a blood pressure cuff to suprasystolic pressures (60 mmHg above the baseline systolic blood pressure) around the brachial artery for 5 min. After the cuff was deflated, the system measured the digital pulse amplitude to capture the post-occlusion response. The log-transformed reactive hyperemia index (Ln-RHI) was calculated by comparing the post-occlusion pulse amplitude with the baseline measurement. The resulting values were reported in standardized arbitrary units, with a predefined Ln-RHI cutoff of <0.51 used to identify the presence of peripheral endothelial dysfunction. This cutoff has been previously validated as an indicator of impaired endothelial function in RA patients. Through this methodology, we identified peripheral ED in patients with Ln-RHI values below the threshold of 0.51, contributing to the understanding of the extent of endothelial dysfunction present in the study population. This assessment of endothelial function allowed for a direct evaluation of vascular health in relation to circulating biomarkers, including Humanin, GAS5, and miRNAs, which were further analyzed for their associations with ED outcomes in this study.

### 4.6. Statistical Analysis

All experiments were conducted in duplicate, with three technical replicates for each treatment. For statistical comparisons, the Kruskal–Wallis test, one-way analysis of variance (ANOVA) with Tukey’s correction, and Wilcoxon tests were applied, considering a *p*-value < 0.05 as statistically significant. Descriptive statistics were used to summarize the data. Qualitative variables were presented as absolute numbers and relative frequencies. Continuous variables were expressed as either the mean with standard deviation or the median with interquartile range (IQR), depending on the normality of the distribution, which was assessed using the Shapiro–Wilk test. Comparisons between groups for qualitative variables were performed using the χ^2^ test, or Fisher’s exact test when cell counts were below 10. For quantitative variables, Student’s t-test or the Mann–Whitney test was used for comparisons between two groups, based on parametric or non-parametric distribution. For comparisons among three or more groups, ANOVA or Kruskal–Wallis tests were employed. Post hoc analyses following the Kruskal–Wallis test were performed using Tukey’s test to identify specific differences between groups and compare outcomes across multiple categories. Significant variables from the univariate analysis were further assessed through bivariate logistic regression. Subsequently, multivariate logistic regression was conducted to account for potential confounding factors. The analyses were performed using Stata software version 14 (StataCorp, College Station, TX, USA) and MedCalc for Windows, version 12.5 64 bit (MedCalc Software, Ostend, Belgium).

## 5. Conclusions

This study demonstrates that Humanin plays a key role in regulating endothelial function and holds potential as a prognostic biomarker for cardiovascular outcomes and survival in RA patients. Higher circulating Humanin levels were significantly associated with improved endothelial function and better survival rates, suggesting its protective role against cardiovascular dysfunction in RA. While non-coding RNAs such as GAS5, miR-21, and miR-103 did not show significant associations with endothelial dysfunction in this study, further investigation with larger cohorts is warranted. Overall, Humanin may be a valuable target for improving cardiovascular health in RA patients, with potential clinical implications in mitigating disease progression and related complications.

## Figures and Tables

**Table 1:** Clinical, demographic, and laboratory variables in rheumatoid arthritis patients vs. controls. RA patients are stratified by cardiovascular risk: low cardiovascular risk as RA-LR, medium cardiovascular risk as RA-MR, high cardiovascular risk as RA-HR, and adverse cardiovascular outcomes as RA-D.

Variable		RA-LR (*n* = 24)	RA-MR (*n* = 25)	RA-HR (*n* = 25)	RA-D (*n* = 16)	Tot (*n* = 71)	*p*
Sex = 1, *n* (%)		0 (0)	2 (8)	9 (36)	8 (50)	19 (21.11)	<0.001
Age, mean (SD)		55.79 (5.52)	66.16 (5.12)	73.08 (8.07)	73.38 (9.80)	66.96 (10.28)	<0.001
stroke, *n* (%)		1 (4.17)	0 (0)	2 (9.09)	0 (0)	3 (3.49)	0.31
tia, *n* (%)		0 (0)	1 (4)	0 (0)	0 (0)	1 (1.16)	1.00
ami, *n* (%)		0 (0)	0 (0)	0 (0)	1 (6.67)	1 (1.16)	0.17
hf, *n* (%)		0 (0)	0 (0)	0 (0)	1 (6.67)	1 (1.16)	0.17
cvd_death, *n* (%)		0 (0)	0 (0)	0 (0)	1 (6.67)	2 (13.33)	0.029
death, *n* (%)		0 (0)	1 (4)	2 (9.09)	16 (100)	19 (21.84)	<0.001
Kg, median (IQR)		60 (56–69)	62 (57–60)	75 (65–80)	68 (60–75)	66 (59.5–75)	0.0034
Cm, mean (SD)		160.48 (5.19)	160.68 (8.52)	163.04 (10.14)	162.13 (7.85)	161.55 (8.15)	0.67
Waist, mean (SD)		91.35 (10.55)	92.67 (14.91)	101.56 (10.65)	95.6 (9.65)	93.38 (12.15)	0.014
Sbp, mean (SD)		116.04 (12.76)	127.4 (14.3)	143.12 (17.11)	137.87 (18.33)	130.52 (18.60)	<0.001
Dbp, mean (SD)		70.29 (11.06)	76.72 (9.14)	78.4 (10.27)	77.73 (9.09)	75.63 (10.38)	0.0261
htndrugs, *n* (%)		4 (17.39)	6 (24)	13 (52)	9 (60)	32 (36.36)	0.01
Chol, mean (SD)		5.32 (0.81)	5.47 (0.88)	5.03 (1.13)	5.35 (1.21)	5.29 (1.00)	0.45
Hdl, median (IQR)		1.75 (1.46–1.94)	1.6 (1.4–1.89)	1.58 (1.19–1.78)	1.31 (1.1–1.76)	1.58 (1.34–1.86)	0.0602
Trigl, median (IQR)		76 (59–94)	82 (59–114)	95 (69–105)	115 (82.5–127)	92 (62–114)	0.0792
Ldl, mean (SD)		125.42 (28.27)	127.36 (31.12)	115.6 (39.93)	121.94 (37.74)	122.61 (34.06)	0.64
Disldrugs, *n* (%)		0 (0)	5 (20)	8 (32)	2 (13.33)	15 (17.05)	0.016
Diabetes, *n* (%)		0 (0)	4 (16)	15 (60)	0 (0)	19 (21.11)	<0.001
smoker, *n* (%)		1 (4.35)	1 (4)	6 (24)	1 (6.67)	9 (10.23)	0.097
acpa, *n* (%)		11 (55)	15 (75)	12 (66.67)	6 (54.55)	44 (63.77)	0.534
rf, *n* (%)		15 (75)	19 (86.36)	20 (90.91)	11 (84.62)	65 (84.42)	0.56
Tjc, median (IQR)		2 (1–6)	2 (1–10)	2 (2–6)	2 (1–3)	2 (1–6)	0.76
Esr, median (IQR)		32 (16–37)	26.5 (14–44.5)	26.5 (13.5–48.5)	44 (20–55.5)	61 (16–47)	0.45
Crpdl, median (IQR)		0.33 (0.1–0.7)	0.37 (0.2–0.84)	0.71 (0.22–0.9)	0.3 (0.19–0.98)	0.38 (0.2–0.9)	0.74
Hb, mean (SD)		12.44 (1.08)	12.92 (1.65)	12.68 (1.66)	13.57 (1.45)	12.85 (1.5)	0.14
Gh, mean (SD)		46.46 (17.35)	52 (23.04)	44 (20.21)	55.71 (26.52)	48.77 (21.48)	0.32
Pgacm, mean (SD)		4.54 (2.30)	5.25 (2.66)	4.28 (2.51)	4.93 (2.73)	4.72 (2.52)	0.57
Egacm, median (IQR)		1.5 (1–2)	2 (1–4)	1 (0–3)	2 (1–5)	2 (1–3)	0.28
Haq, median (IQR)		0.38 (0–1)	0.88 (0.32–1)	1 (0.25–1.25)	1 (0.5–1.87)	0.94 (0.13–1)	0.10
Steroid, *n* (%)		9 (37.5)	10 (41.67)	9 (36)	6 (42.86)	34 (39.08)	0.97
Steroidmgday, median (IQR)		0 (0–3.5)	0 (0–5)	0 (0–3.5)	0 (0–3)	0 (0–4)	0.84
Cumulative, median (IQR)		0 (0–97.5)	0 (0–150)	0 (0–75)	0 (0–80)	0 (0–120)	0.89
Nsaids, *n* (%)		4 (16.67)	6 (25)	6 (24)	4 (28.57)	20 (22.99)	0.866
Freqnsaids, median (IQR)		0 (0–0)	0 (0–1)	0 (0–0)	0 (0–1)	0 (0–0)	0.87
dmards, *n* (%)		16 (66.67)	14 (58.33)	22 (88)	8 (57.14)	60 (68.97)	0.072
numberofdmards	1, *n* (%)	11 (45.83)	9 (37.5)	18 (72)	6 (42.86)	44 (50.57)	0.197
	2, *n* (%)	5 (20.83)	5 (20.83)	4 (16)	2 (14.29)	16 (18.39)	
Mtx, *n* (%)		16 (66.67)	11 (45.83)	15 (60)	7 (50)	49 (56.32)	0.479
Mtxdose, median (IQR)		10 (0–15)	0 (0–15)	10 (0–15)	5 (0–15)	10 (0–15)	0.67
hcq, *n* (%)		4 (16.67)	6 (25)	10 (40)	2 (14.29)	22 (25.29)	0.195
hcqdose, median (IQR)		0 (0–0)	0 (0–100)	0 (0–200)	0 (0-0)	0 (0–200)	0.25
tnfi, *n* (%)		9 (37.5)	5 (20.83)	3 (12)	4 (28.57)	21 (24.14)	0.2
toci, *n* (%)		0 (0)	1 (4.17)	1 (4)	0 (0)	2 (2.3)	0.66
aba, *n* (%)		1 (4.17)	1 (4.17)	1 (4)	0 (0)	3 (3.45)	0.897
Lnrhi, median (IQR)		0.99 (0.89–1.16)	1.08 (0.98–1.16)	1.17 (0.69–1.33	0.27 (0.15–0.37)	0.99 (0.72–1.18)	<0.001

Legend for abbreviations used in Table 1: RA-LR: rheumatoid arthritis with low cardiovascular risk; RA-MR: rheumatoid arthritis with medium cardiovascular risk; RA-HR: rheumatoid arthritis with high cardiovascular risk; RA-D: rheumatoid arthritis with adverse cardiovascular outcomes; *n* (%): number and percentage of subjects; SD: standard deviation; IQR: interquartile range; Kg: weight in kilograms; Cm: height in centimeters; Sbp: systolic blood pressure; Dbp: diastolic blood pressure; htndrugs: antihypertensive drugs; Chol: total cholesterol; Hdl: high-density lipoprotein cholesterol; Trigl: triglycerides; Ldl: low-density lipoprotein cholesterol; Disldrugs: lipid-lowering drugs; acpa: anti-citrullinated protein antibodies; rf: rheumatoid factor; Tjc: tender joint count; Esr: erythrocyte sedimentation rate; Crpdl: C-reactive protein levels; Hb: hemoglobin; Gh: General Health Assessment; Pgacm: Patient Global Assessment (Centimeter Visual Analog Scale); Egacm: Evaluator Global Assessment (Centimeter Visual Analog Scale); Haq: Health Assessment Questionnaire Score; Steroid: use of steroids; Steroidmgday: steroid dose in mg/day; Nsaids: nonsteroidal anti-inflammatory drugs; dmards: disease-modifying antirheumatic drugs; Mtx: Methotrexate; hcq: Hydroxychloroquine; tnfi: tumor necrosis factor inhibitors; toci: Tocilizumab; aba: Abatacept; Lnrhi: logarithmic reactive hyperemia index (measure of endothelial function).

**Table 2:** Logistic regression analysis of biomarkers and cardiovascular risk (SCORE-2): Evaluation of association between various biomarkers (Humanin, miR-21, miR-103, and GAS5, both in plasma and exosomes) and endothelial dysfunction, accounting for additional variables including age, sex, and SCORE2_EULAR score. “Odds ratio” and “95% CI” values offer estimates of relative likelihood of endothelial dysfunction associated with each biomarker.

Variable	95% CI	Odds Ratio	*p* Value
*HUMANIN_pg_ml*	*0.96 to 1*	0.98	0.02
age	0.94 to 1.12	1.03	0.57
sex	0.15 to 2.54	0.62	0.5
SCORE2_EULAR	0 to 4.29	0.02	0.15
Variable	95% CI	Odds ratio	*p* value
*miR_21*	*0.85 to 1.22*	1.02	0.83
age	0.98 to 1.15	1.07	0.13
sex	0.16 to 2.27	0.59	0.45
SCORE2_EULAR	0 to 0.48	0	0.03
Variable	95% CI	Odds ratio	*p* value
*miR_21_Exosomes*	*0.85 to 1.44*	1.11	0.45
age	0.98 to 1.15	1.06	0.15
sex	0.15 to 2.2	0.57	0.41
SCORE2_EULAR	0 to 0.51	0	0.03
Variable	95% CI	Odds ratio	*p* value
*miR_103*	*0.67 to 1.32*	0.94	0.72
age	0.98 to 1.16	1.07	0.11
sex	0.16 to 2.4	0.62	0.49
SCORE2_EULAR	0 to 0.48	0	0.03
Variable	95% CI	Odds ratio	*p* value
*miR_103_Exosomes*	*0.57 to 2.03*	1.07	0.83
age	0.98 to 1.16	1.07	0.12
sex	0.15 to 2.26	0.59	0.44
SCORE2_EULAR	0 to 0.47	0	0.03
Variable	95% CI	Odds ratio	*p* value
*GAS5*	*0.91 to 1.32*	1.1	0.33
age	0.99 to 1.17	1.07	0.09
sex	0.15 to 2.29	0.59	0.45
SCORE2_EULAR	0 to 0.43	0	0.02
Variable	95% CI	Odds ratio	*p* value
*GAS5_Exosomes*	*0.92 to 1.38*	1.13	0.24
age	0.99 to 1.16	1.07	0.09
sex	0.18 to 2.91	0.72	0.65
SCORE2_EULAR	0 to 1.1	0.01	0.05

**Table 3:** ROC curve analysis for humanin levels and prediction of endothelial dysfunction.

Metric	Value
**Area under ROC Curve (AUC)**	0.68
Standard Error	0.06
95% Confidence Interval	0.57 to 0.78
z Statistic	3.17
Significance Level (*p*) (Area = 0.5)	0.0015
Youden Index (J)	0.41
Associated Criterion	≤124.44 pg/mL

**Table 4:** Kaplan–Meier survival analysis and hazard ratio for humanin levels.

Metric	Value
**Endpoint: Observed (*n*)**	3 (Low Humanin)/16 (High Humanin)
Expected (*n*)	14.8/4.2
Chi-square	42.56
Degrees of Freedom (DF)	1
Significance	*p* < 0.0001
Hazard Ratio	18.66
95% Confidence Interval (CI)	6.33 to 55.02

**Table 5:** Kaplan–Meier survival information with the number of individuals at risk stratified by time.

Time	Number at Risk (Group 0)	Number at Risk (Group 1)
0	71	16
500	71	15
1000	70	11
1500	67	11
2000	42	4
2500	0	0

**Table 6:** miRNA accession numbers, symbols, and sequences.

Accession ID Number	Symbol	Sequence
MI0000077	hsa-miR 21-5p	UAGCUUAUCAGACUGAUGUUGA
MI0000109	hsa-miR-103a-3p	AGCAGCAUUGUACAGGGCUAUGA

**Table 7:** Primer sequences.

Primer Name	Forward	Reverse
hGAPDH	GAGTCAACGGAATTTGGTCGT	GACAAGCTTCCCGTTCTCAG
GAS5	5′-CTTGCCTGGACCAGCTTAAT-3′	5′-CAAGCCGACTCTCCATACCT-3′

## Data Availability

Relevant data are included in the main body of this manuscript. All other information is available on request.

## References

[1] Wu D., Luo Y., Li T., Zhao X., Lv T., Fang G., Ou P., Li H., Luo X., Huang A. (2022). Systemic complications of rheumatoid arthritis: Focus on pathogenesis and treatment. Front. Immunol..

[2] Gao Y., Zhang Y., Liu X. (2024). Rheumatoid arthritis: Pathogenesis and therapeutic advances. MedComm.

[3] Kim J.-W., Suh C.-H. (2020). Systemic manifestations and complications in patients with rheumatoid arthritis. J. Clin. Med..

[4] Arida A., Protogerou A.D., Kitas G.D., Sfikakis P.P. (2018). Systemic inflammatory response and atherosclerosis: The paradigm of chronic inflammatory rheumatic diseases. Int. J. Mol. Sci..

[5] Özkan U., Kakilli N., Gürdoğan M., Taştekin N., Birtane M. (2023). Rheumatoid arthritis and cardiovascular comorbidities. Explor. Musculoskelet. Dis..

[6] Peyronnel C., Totoson P., Martin H., Demougeot C. (2023). Relevance of circulating markers of endothelial activation for cardiovascular risk assessment in rheumatoid arthritis: A narrative review. Life Sci..

[7] Sandoo A., Carroll D., Metsios G.S., Kitas G.D., Veldhuijzen van Zanten J.J. (2011). The association between microvascular and macrovascular endothelial function in patients with rheumatoid arthritis: A cross-sectional study. Arthritis Res. Ther..

[8] Sun H.-J., Wu Z.-Y., Nie X.-W., Bian J.-S. (2020). Role of endothelial dysfunction in cardiovascular diseases: The link between inflammation and hydrogen sulfide. Front. Pharmacol..

[9] Medina-Leyte D.J., Zepeda-García O., Domínguez-Pérez M., González-Garrido A., Villarreal-Molina T., Jacobo-Albavera L. (2021). Endothelial dysfunction, inflammation and coronary artery disease: Potential biomarkers and promising therapeutical approaches. Int. J. Mol. Sci..

[10] Johri N., Varshney S., Gandha S., Maurya A., Mittal P., Jangra S., Garg R., Saraf A. (2023). Association of cardiovascular risks in rheumatoid arthritis patients: Management, treatment and future perspectives. Health Sci. Rev..

[11] Coradduzza D., Bo M., Congiargiu A., Azara E., De Miglio M.R., Erre G.L., Carru C. (2023). Decoding the microbiome’s influence on rheumatoid arthritis. Microorganisms.

[12] Oh Y.K., Bachar A.R., Zacharias D.G., Kim S.G., Wan J., Cobb L.J., Lerman L.O., Cohen P., Lerman A. (2011). Humanin preserves endothelial function and prevents atherosclerotic plaque progression in hypercholesterolemic ApoE deficient mice. Atherosclerosis.

[13] Widmer R.J., Flammer A., Herrmann J., Rodriguez-Porcel M., Wan J., Cohen P., Lerman L.O., Lerman A. (2013). Circulating humanin levels are associated with preserved coronary endothelial function. Am. J. Physiol. Heart Circ. Physiol..

[14] Coradduzza D., Congiargiu A., Chen Z., Cruciani S., Zinellu A., Carru C., Medici S. (2023). Humanin and its pathophysiological roles in aging: A systematic review. Biology.

[15] Cai H., Liu Y., Men H., Zheng Y. (2021). Protective mechanism of humanin against oxidative stress in aging-related cardiovascular diseases. Front. Endocrinol..

[16] Peng T., Ji D., Jiang Y. (2021). Long Non-Coding RNA GAS5 Suppresses Rheumatoid Arthritis Progression via miR-128-3p/HDAC4 Axis. Mol. Cell. Biochem..

[17] Kirkman D.L., Robinson A.T., Rossman M.J., Seals D.R., Edwards D.G. (2021). Mitochondrial contributions to vascular endothelial dysfunction, arterial stiffness, and cardiovascular diseases. Am. J. Physiol. -Heart Circ. Physiol..

[18] Yamamura S., Imai-Sumida M., Tanaka Y., Dahiya R. (2018). Interaction and cross-talk between non-coding RNAs. Cell. Mol. Life Sci..

[19] Coradduzza D., Bellu E., Congiargiu A., Pashchenko A., Amler E., Necas A., Carru C., Medici S., Maioli M. (2022). Role of nano-mirnas in diagnostics and therapeutics. Int. J. Mol. Sci..

[20] Li Y. (2023). Non–Coding RNA Performs Its Biological Function by Interacting with Macromolecules. Int. J. Mol. Sci..

[21] Huang H., Li L., Wen K. (2021). Interactions between long non-coding RNAs and RNA-binding proteins in cancer. Oncol. Rep..

[22] Scholda J., Nguyen T.T.A., Kopp F. (2024). Long noncoding RNAs as versatile molecular regulators of cellular stress response and homeostasis. Hum. Genet..

[23] Fernández-Hernando C., Suárez Y. (2018). MicroRNAs in endothelial cell homeostasis and vascular disease. Curr. Opin. Hematol..

[24] Khalaji A., Mehrtabar S., Jabraeilipour A., Doustar N., Rahmani Youshanlouei H., Tahavvori A., Fattahi P., Alavi S.M.A., Taha S.R., Fazlollahpour-Naghibi A. (2024). Inhibitory effect of microRNA-21 on pathways and mechanisms involved in cardiac fibrosis development. Ther. Adv. Cardiovasc. Dis..

[25] Huang Y. (2018). The novel regulatory role of lnc RNA-mi RNA-mRNA axis in cardiovascular diseases. J. Cell. Mol. Med..

[26] Li M., Wang N., Shen Z., Yan J. (2020). Long Non-Coding RNA Growth Arrest-Specific Transcript 5 Regulates Rheumatoid Arthritis via Targeting Homeodomain-Interacting Protein Kinase 2. Clin. Exp. Rheumatol..

[27] Drosos G.C., Vedder D., Houben E., Boekel L., Atzeni F., Badreh S., Boumpas D.T., Brodin N., Bruce I.N., González-Gay M.Á. (2022). EULAR recommendations for cardiovascular risk management in rheumatic and musculoskeletal diseases, including systemic lupus erythematosus and antiphospholipid syndrome. Ann. Rheum. Dis..

[28] Erre G.L., Chessa I., Bassu S., Cavagna L., Carru C., Pintus G., Giordo R., Mangoni A.A., Damiano Sanna G., Zinellu A. (2024). Association between ischemia-modified albumin (IMA) and peripheral endothelial dysfunction in rheumatoid arthritis patients. Sci. Rep..

[29] Montes D., Hulshizer C.A., Myasoedova E., Davis J.M., Hanson A.C., Duarte-Garcia A., Figueroa-Parra G., Chevet B., Crowson C.S. (2023). Utilisation of cardiovascular preventive services in a rheumatoid arthritis population-based cohort. RMD Open.

